# Supplementary material for: High-precision spatial analysis of mouse courtship vocalization behavior reveals sex and strain differences
Source: Sci Rep. 2023 Mar 30;13:5219. doi: 10.1038/s41598-023-31554-3 (PMC10063627; doi:10.1038/s41598-023-31554-3)
Supplement: Supplementary file 1 — Supplementary Legends. [file 41598_2023_31554_MOESM1_ESM.docx]

**Movie 1:** Short example video that shows automatic tracking results of two behaving mice in the experimental setup (red markers: female mouse, blue markers: male mouse). Top row on right-hand side shows corresponding microphone recordings in the ultrasonic range.

**Movie 2:** Same data as in Figure 6 revolving in 3D to resolve depth ambiguities. Dimensionality reduction of USV properties detects relations between phonetics, strain, sex, and relative position.

**A** Each USV was represented by its main frequency line (up to 100 ms), its derivative, the duration, the directionality in frequency, and the snout-to-snout distance (203 dimensions) and then reduced to 3 dimensions using UMAP [(McInnes et al., 2018)](https://sciwheel.com/work/citation?ids=5973004&pre=&suf=&sa=0). The analysis was based on the set of selected vocalizations (see Fig. 4). The results are differently color-coded for each property.

**B** We evaluated whether a property of the USV was related to others after dimensionality reduction by comparing the original (left) to shuffled data (right; same spatial and value distribution) using nearest neighbor decoding. This allowed us to assess the percentage of variance explained as the relation between the local prediction error (LPE, see *Methods* for details) of the original and shuffled data.

**C** After dimensionality reduction, the set of spectrograms exhibited a rich structure. We highlight the richness of the substructure here by running clustering (k-means, k=100, different colors). However, clusters are often not clearly separated but rather connected. The properties in the following panels partially clarify the origin of this substructure.

**D** Sex showed a significant contribution to accounting for the neighborhood structure, shown here via local density differences between male and female emitters. While significant, these differences explain only 3.4% of the LPE.

**E** The Foxp2-R552H variant (male vocalizers only) also significantly accounted for the local structure, accounting for 9.8% of the LPE. Interestingly, differences in spatial density between the strains (Foxp2-R552H: orange; WT littermate: green) partly coincided with the sex differences in **D**.

**F** Among the tested properties, duration best explained the neighborhood structure of the spectrograms by being able to account for 81% of the LPE.

**G** Mean frequency also explained a substantial part of the neighborhood structure, although largely locally 'orthogonal' to the duration (i.e. multiple local gradients of frequency), accounting for 72% of the LPE.

**H** Frequency range explained 30% of the LPE and appears correlated in spatial distribution with duration.

**I** The Wiener Entropy also explained a substantial part of the LPE (37%).

**J** Different snout-snout distances only contributed borderline significantly to the structuring accounting for 1.3% of the LPE. Supplementary Figures 5/6 and Supplemental Movies 3/4 show the same analysis when excluding duration or duration and mean frequency.

**Movie 3:** Data from Supplementary Figure 5 revolving in 3D to resolve depth ambiguities. Same analysis as in Fig. 6, but with duration removed from the analysis by first stretching the USV to the same length as often done in other studies. The explained variance by the Foxp2-R552H variant dropped slightly, consistent with the differences in USV duration in Fig. 6 (N.B., the latter are spatially resolved, while these are partially averaged). The explained variance of the different genotypes increased to 14%, pointing to a variant-specific difference in the shape of the USVs. Note that duration, despite its removal, still had predictive value due to its correlation with other properties, e.g. frequency range.

**Movie 4:** Data from Supplementary Figure 6 revolving in 3D to resolve depth ambiguities. Same analysis as in Fig. 6, but now with duration and mean frequency removed (i.e. all vocalizations centered on the same frequency). As a consequence, the variance that duration and mean frequency explain ends up substantially lower while frequency range slightly increases. The explained variance by genotype is still low, indicating that frequency differences are contributing to the genotype differences in vocalization.
