# Supplementary material for: High-precision spatial analysis of mouse courtship vocalization behavior reveals sex and strain differences
Source: Sci Rep. 2023 Mar 30;13:5219. doi: 10.1038/s41598-023-31554-3 (PMC10063627; doi:10.1038/s41598-023-31554-3)
Supplement: Supplementary file 6 — Supplementary Figure 1. [file 41598_2023_31554_MOESM6_ESM.docx]

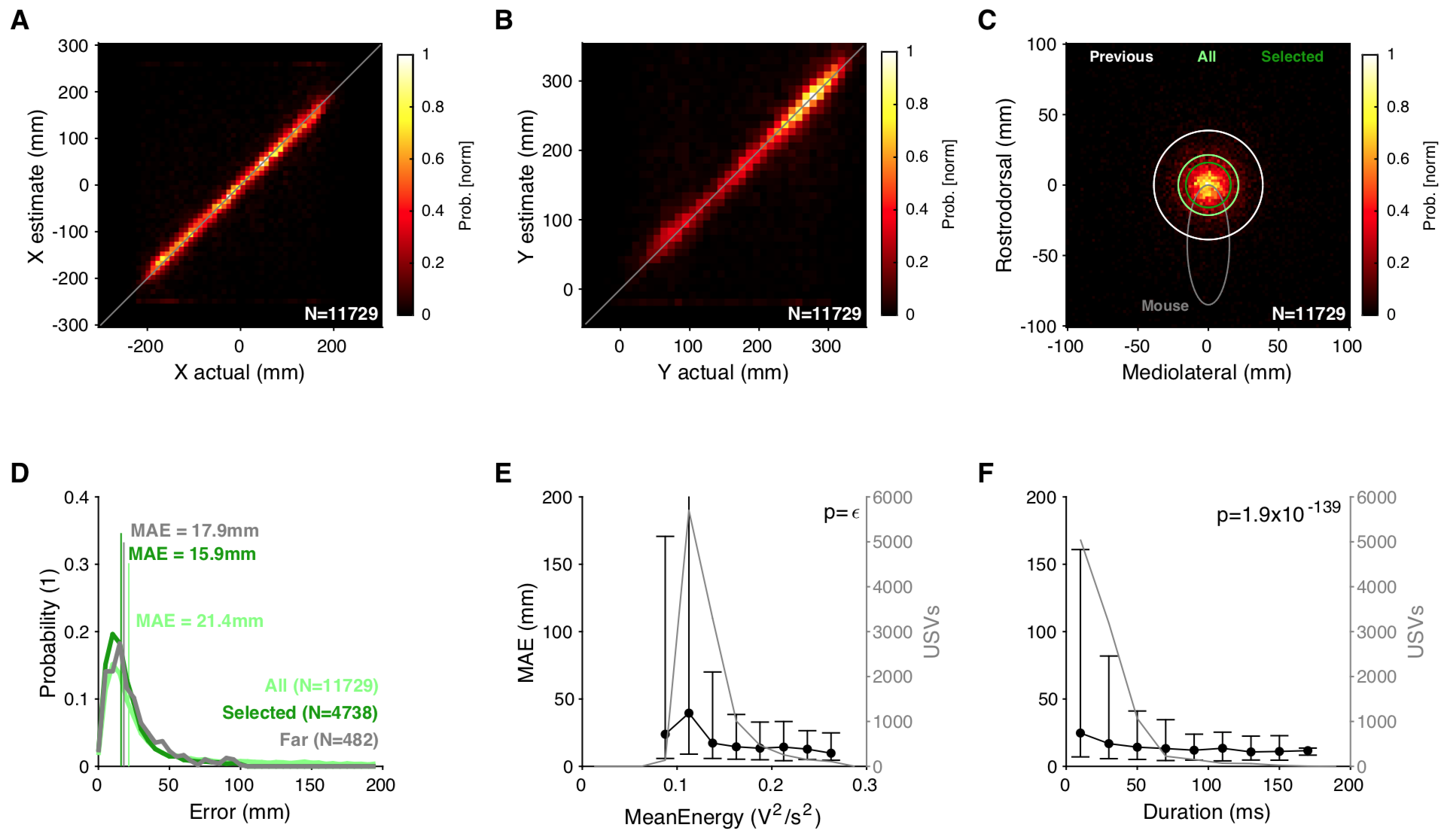


**Supplementary Figure 1:** Supporting data for Figure 3. SLIM still provides high quality localization of vocalizations for 3 microphones. In Exp. 2, CBA/CaJ WT mice were recorded during social interaction using 3 microphones (located in an isosceles triangle nearly enclosing the platform in area, with the front two microphones in the same location as in Exp. 1 and the third one behind it in the middle), the minimal number of microphone necessary for SLIM to work. The results are comparable overall but less accurate than with 4 microphones (**A-C**), i.e. with an MAE = 21.4mm and 15.9 mm for all and the selected (see *Methods* for criteria) USVs, respectively. The percentage of reliably assignable USVs is substantially lower at 40.4%. (**D**). The dependence on mean energy (**E**) and duration (**F**) had a similar shape as for 4 microphones.
