# Supplementary material for: High-precision spatial analysis of mouse courtship vocalization behavior reveals sex and strain differences
Source: Sci Rep. 2023 Mar 30;13:5219. doi: 10.1038/s41598-023-31554-3 (PMC10063627; doi:10.1038/s41598-023-31554-3)
Supplement: Supplementary file 7 — Supplementary Figure 2. [file 41598_2023_31554_MOESM7_ESM.docx]

**
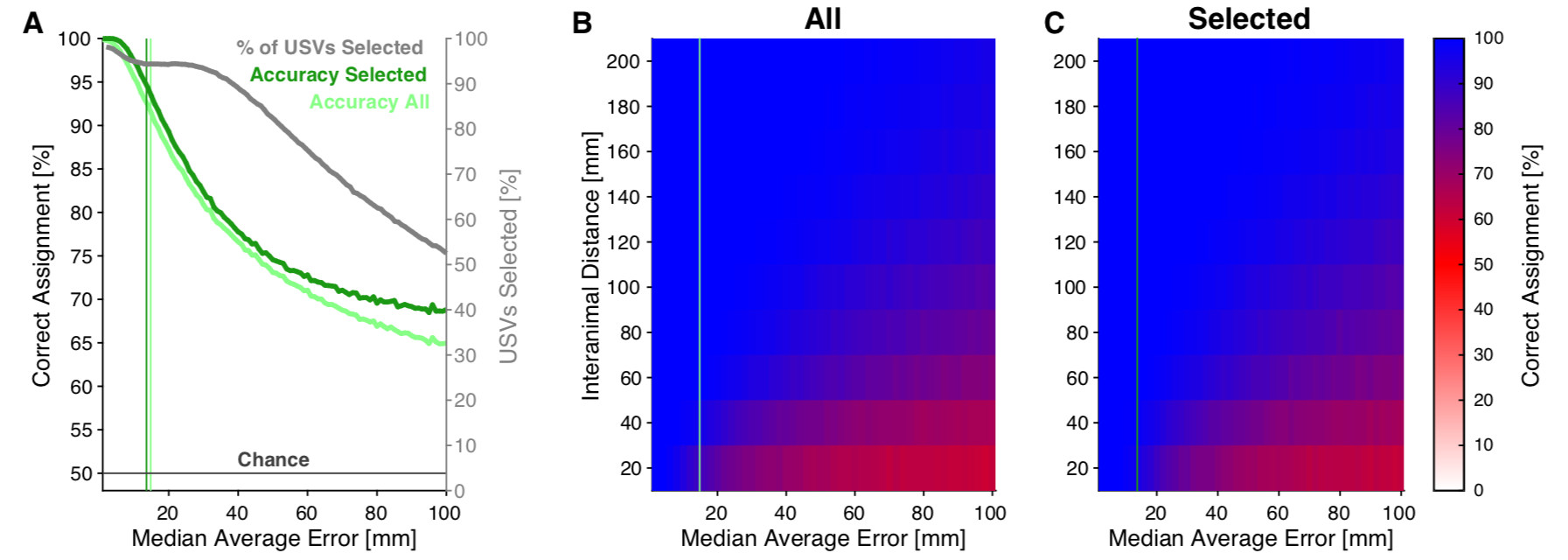
**

**Supplementary Figure 2:** Additional supporting data for Figure 3. Influence of MAE and Selection Criterion on Accuracy of Assignment. We assessed the influence of our selection criteria on the resulting accuracy for different levels of median absolute errors by means of a simulation based on the empirical distance distribution of mice during interaction in our experiment (see Fig. 1D).

**A** The accuracy decreased monotonically for increasing MAEs as expected. Applying our selection criteria (ii) and (iii) (see *Methods*), the accuracy increased substantially, with a widening margin for greater MAEs. The fraction of USVs that passed these criteria decreased strongly with MAE but remained high around the empirical MAE in our study (shown by light and dark green vertical lines, for All and Selected USVs, respectively).

**B/C** In a second simulation we split up these results conditionally on interanimal distance, which demonstrated the expected result that for small interanimal distances the accuracy can be low for high MAEs. However, for the selected (reliably assignable) USVs and given the empirical MAE, the accuracy remains rather high at ~90%, even for close snout-snout contact (range of up to 2cm).
