# Supplementary material for: High-precision spatial analysis of mouse courtship vocalization behavior reveals sex and strain differences
Source: Sci Rep. 2023 Mar 30;13:5219. doi: 10.1038/s41598-023-31554-3 (PMC10063627; doi:10.1038/s41598-023-31554-3)
Supplement: Supplementary file 8 — Supplementary Figure 3. [file 41598_2023_31554_MOESM8_ESM.docx]

**
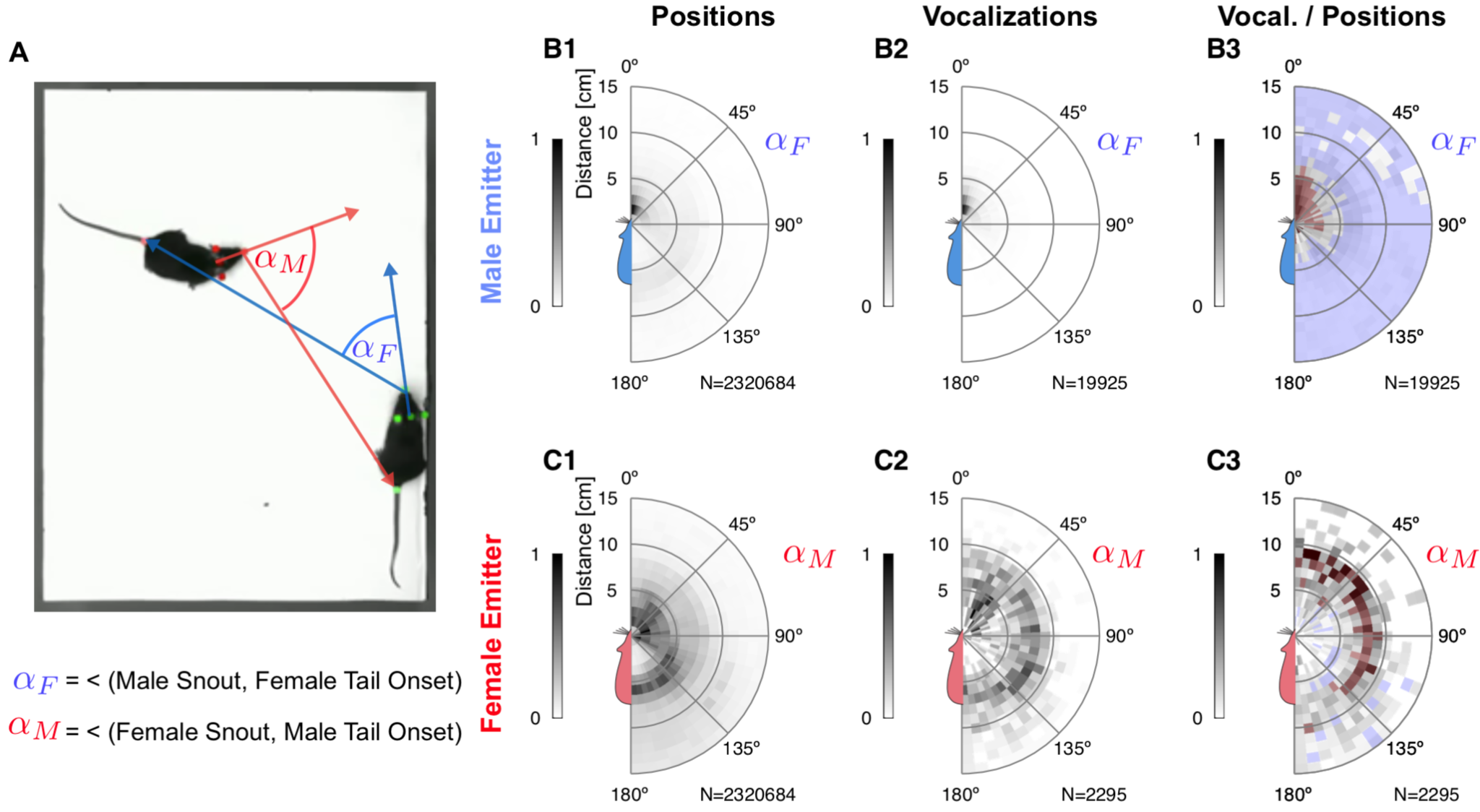
**

**Supplementary Figure 3:** Supporting data for Figure 4. Relative emitter-snout and receiver-tail spatial densities during social interaction. The figure follows the same layout as Figure 4, with the receiver-head replaced by the receiver-tail-onset in the analysis as shown by the arrows in **A**. The males vocalized mainly around the tail-onset location of the female (**B3**), while the females vocalized mostly in snout-snout contexts, where the tail-onset of the male mouse is ~6-10 cm away (**C3**); compare this to Fig. 4C3 as well.
