# Supplementary material for: High-precision spatial analysis of mouse courtship vocalization behavior reveals sex and strain differences
Source: Sci Rep. 2023 Mar 30;13:5219. doi: 10.1038/s41598-023-31554-3 (PMC10063627; doi:10.1038/s41598-023-31554-3)
Supplement: Supplementary file 9 — Supplementary Figure 4. [file 41598_2023_31554_MOESM9_ESM.docx]

**
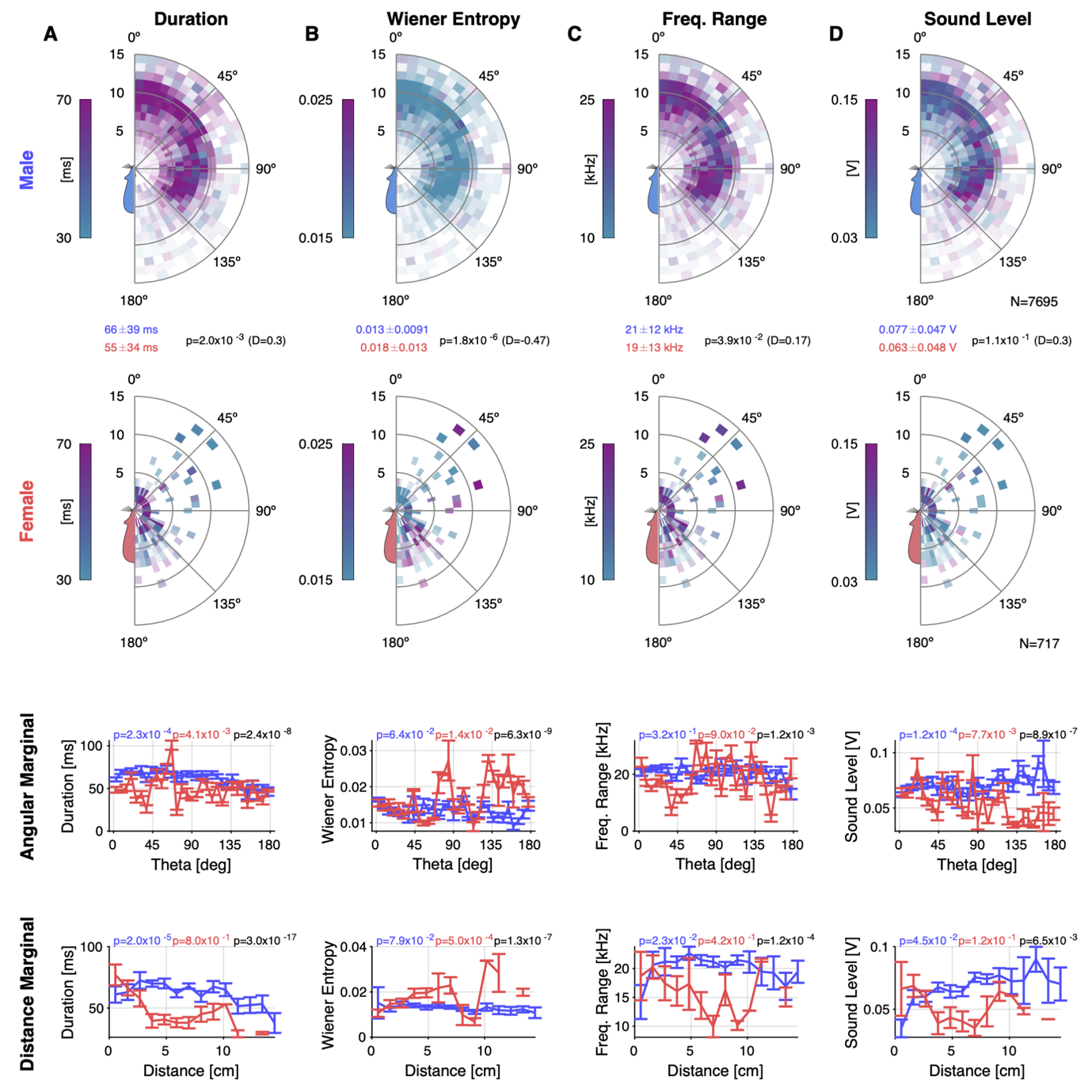
**

**Supplementary Figure 4:** Supporting data for Figure 5. Properties of USV production depend on relative position and sex.

**A** The duration of male USVs was substantially longer for males during snout-anogenital interaction than that of the duration of female USVs (comparison of top two rows). Male USV duration depended on both angle and distance (bottom rows), while female USV duration depended only weakly on these two factors.

**B** The Wiener entropy of female USVs was greater than those of male USVs, in particular during snout-anogenital or snout-snout interactions where the male was behind the female.

**C** The frequency range of male USVs was larger than that of female USVs, and they differed significantly as a function of distance and angle.

**D** While there was no overall difference in sound level between male and female mice (see top), the spatial distributions show different patterns of dependence as a function of distance and angle, with male vocalization levels exceeding those of females for distances >5 cm and angles >100º, indicating snout-anogenital contact with the animals facing in opposite directions.
