# Supplementary material for: High-precision spatial analysis of mouse courtship vocalization behavior reveals sex and strain differences
Source: Sci Rep. 2023 Mar 30;13:5219. doi: 10.1038/s41598-023-31554-3 (PMC10063627; doi:10.1038/s41598-023-31554-3)
Supplement: Supplementary file 10 — Supplementary Figure 5. [file 41598_2023_31554_MOESM10_ESM.docx]

**
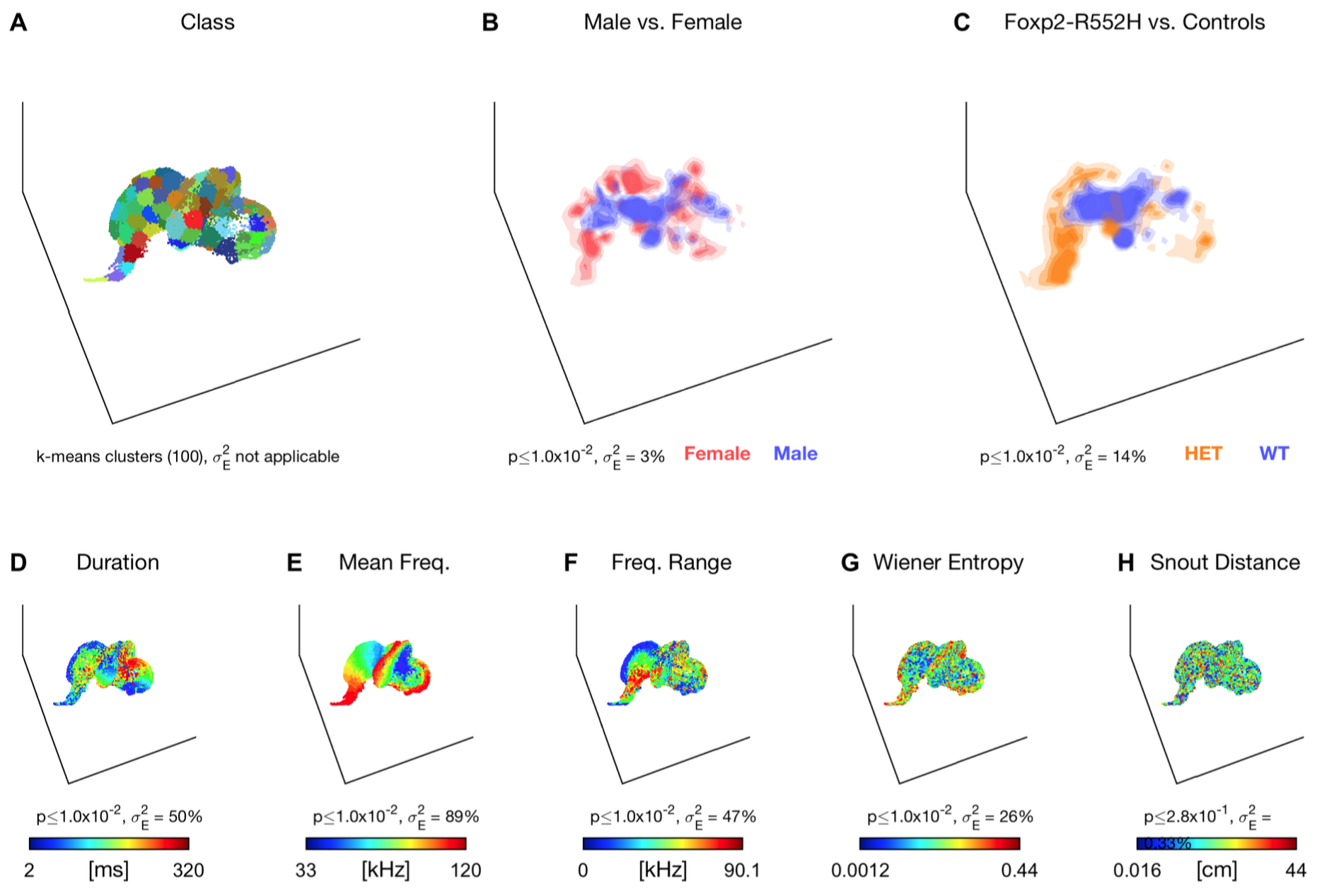
**

**Supplementary Figure 5:** Supporting data for Figure 6. Same analysis as in Fig. 6, but with duration removed from the analysis by first stretching the USV to the same length as often done in other studies. The explained variance by the Foxp2-R552H variant dropped slightly, consistent with the differences in USV duration in Fig. 6 (N.B., the latter are spatially resolved, while these are partially averaged). The explained variance of the different genotypes increased to 14%, pointing to a variant-specific difference in the shape of the USVs. Note that duration, despite its removal, still had predictive value due to its correlation with other properties, e.g. frequency range. Supplemental Movie 3 shows the same data revolving in 3D, resolving depth ambiguities.
