# Supplementary material for: High-precision spatial analysis of mouse courtship vocalization behavior reveals sex and strain differences
Source: Sci Rep. 2023 Mar 30;13:5219. doi: 10.1038/s41598-023-31554-3 (PMC10063627; doi:10.1038/s41598-023-31554-3)
Supplement: Supplementary file 11 — Supplementary Figure 6. [file 41598_2023_31554_MOESM11_ESM.docx]

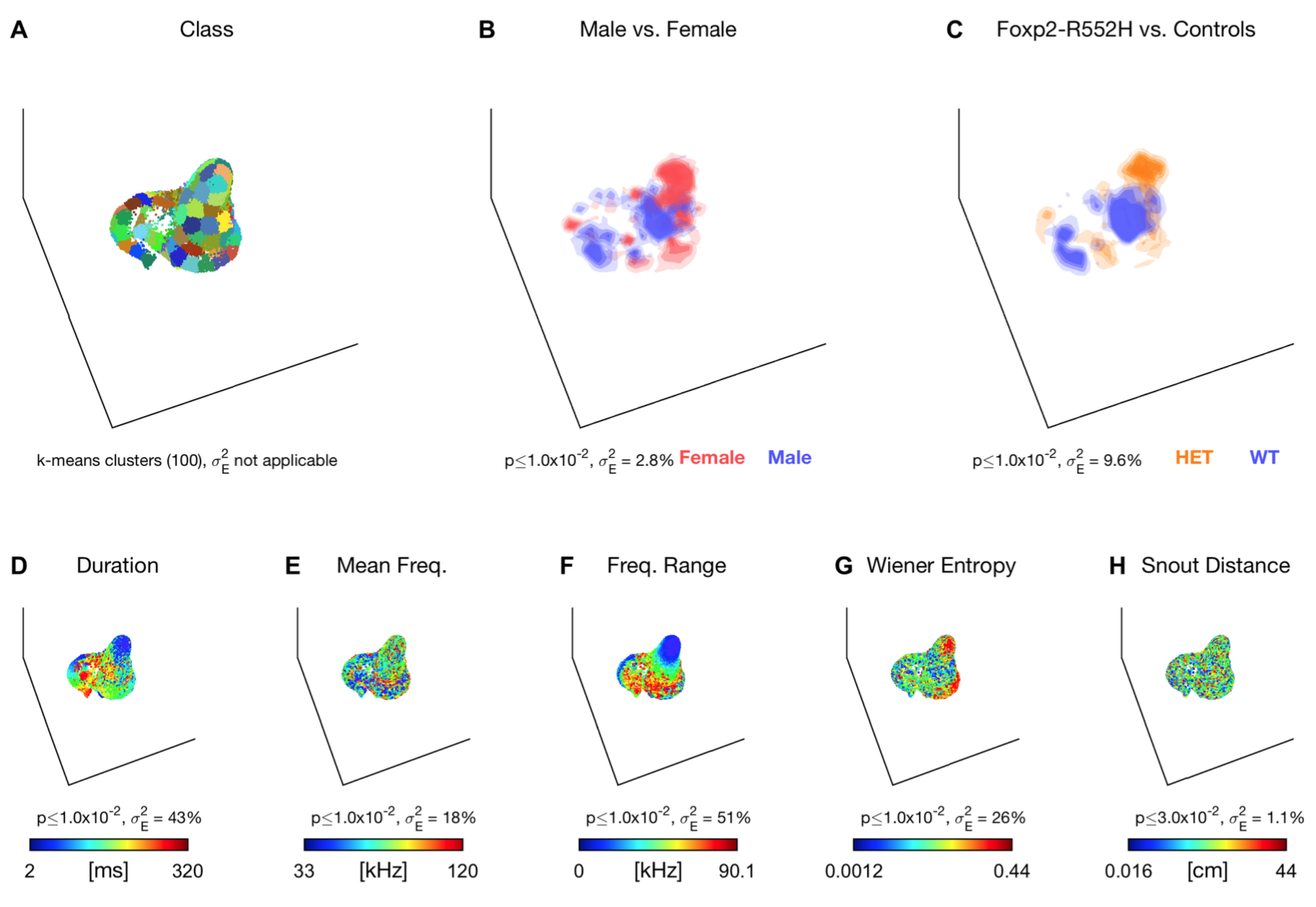


**Supplementary Figure 6:** Additional supporting data for Figure 6. Same analysis as in Fig. 6, but now with duration and mean frequency removed (i.e. all vocalizations centered on the same frequency). As a consequence, the variance that duration and mean frequency explain ends up substantially lower while frequency range slightly increases. The explained variance by genotype is still low, indicating that frequency differences are contributing to the genotype differences in vocalization. Supplemental Movie 4 shows the same data revolving in 3D, resolving depth ambiguities.
