# Supplementary figures and images for: High-precision spatial analysis of mouse courtship vocalization behavior reveals sex and strain differences
Source: Sci Rep. 2023 Mar 30;13:5219. doi: 10.1038/s41598-023-31554-3 (PMC10063627; doi:10.1038/s41598-023-31554-3)

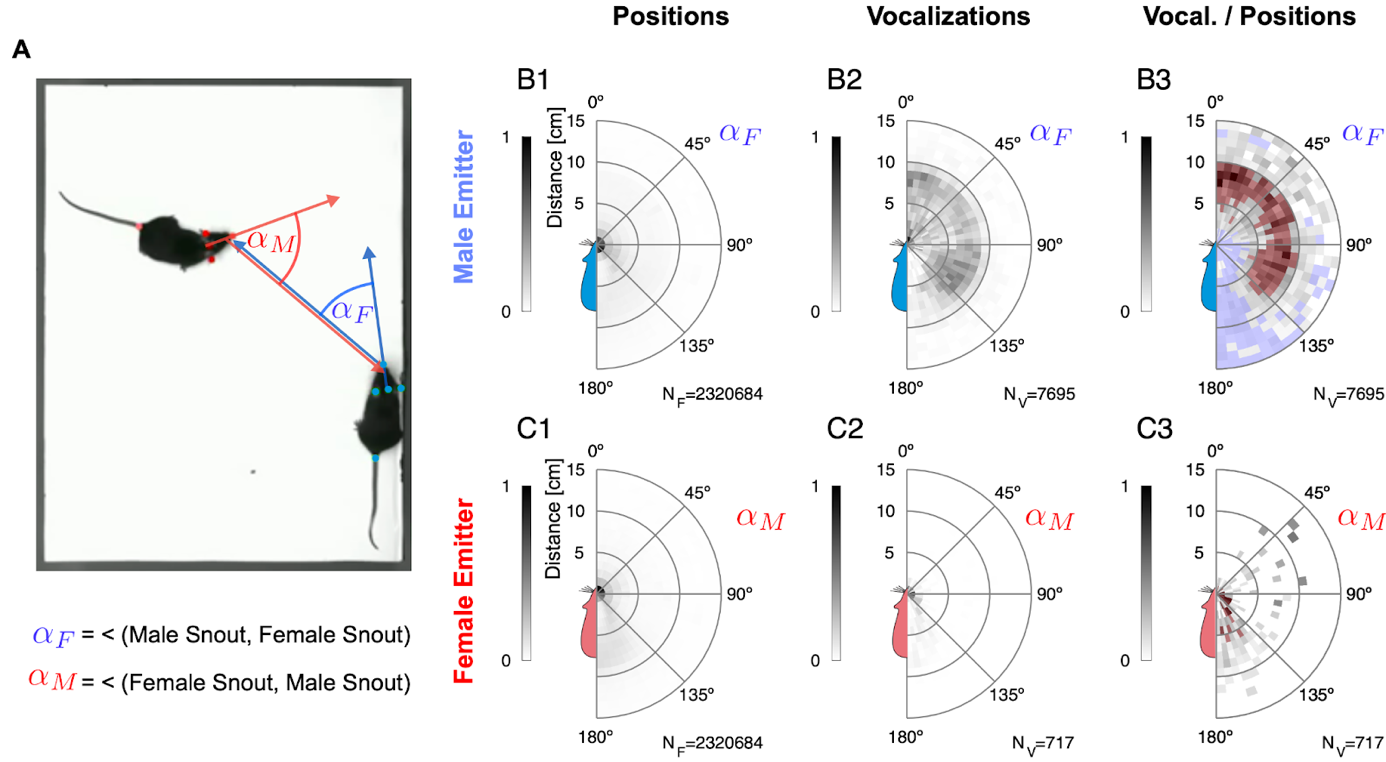


**Supplementary Figure 7:** Same analysis as in Figure 4, for only the WT littermates.

Supplement: Supplementary file 12 — Supplementary Figure 7. [file 41598_2023_31554_MOESM12_ESM.docx]

**
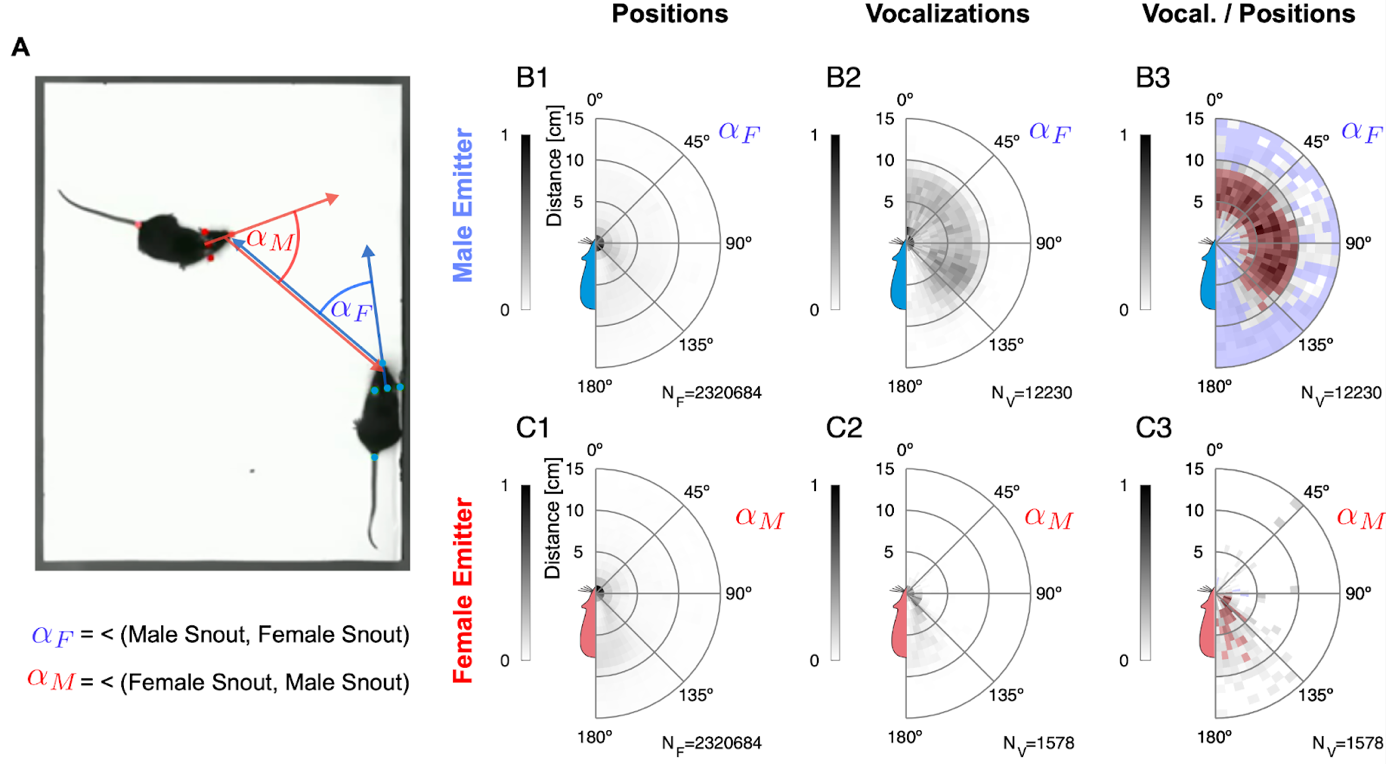
**

**Supplementary Figure 8:** Same analysis as in Figure 4, for only the FoxP2-R552H mice.

Supplement: Supplementary file 13 — Supplementary Figure 8. [file 41598_2023_31554_MOESM13_ESM.docx]
