# Supplementary material for: High-precision spatial analysis of mouse courtship vocalization behavior reveals sex and strain differences
Source: Sci Rep. 2023 Mar 30;13:5219. doi: 10.1038/s41598-023-31554-3 (PMC10063627; doi:10.1038/s41598-023-31554-3)
Supplement: Supplementary file 14 — Supplementary Figure 9. [file 41598_2023_31554_MOESM14_ESM.docx]

**
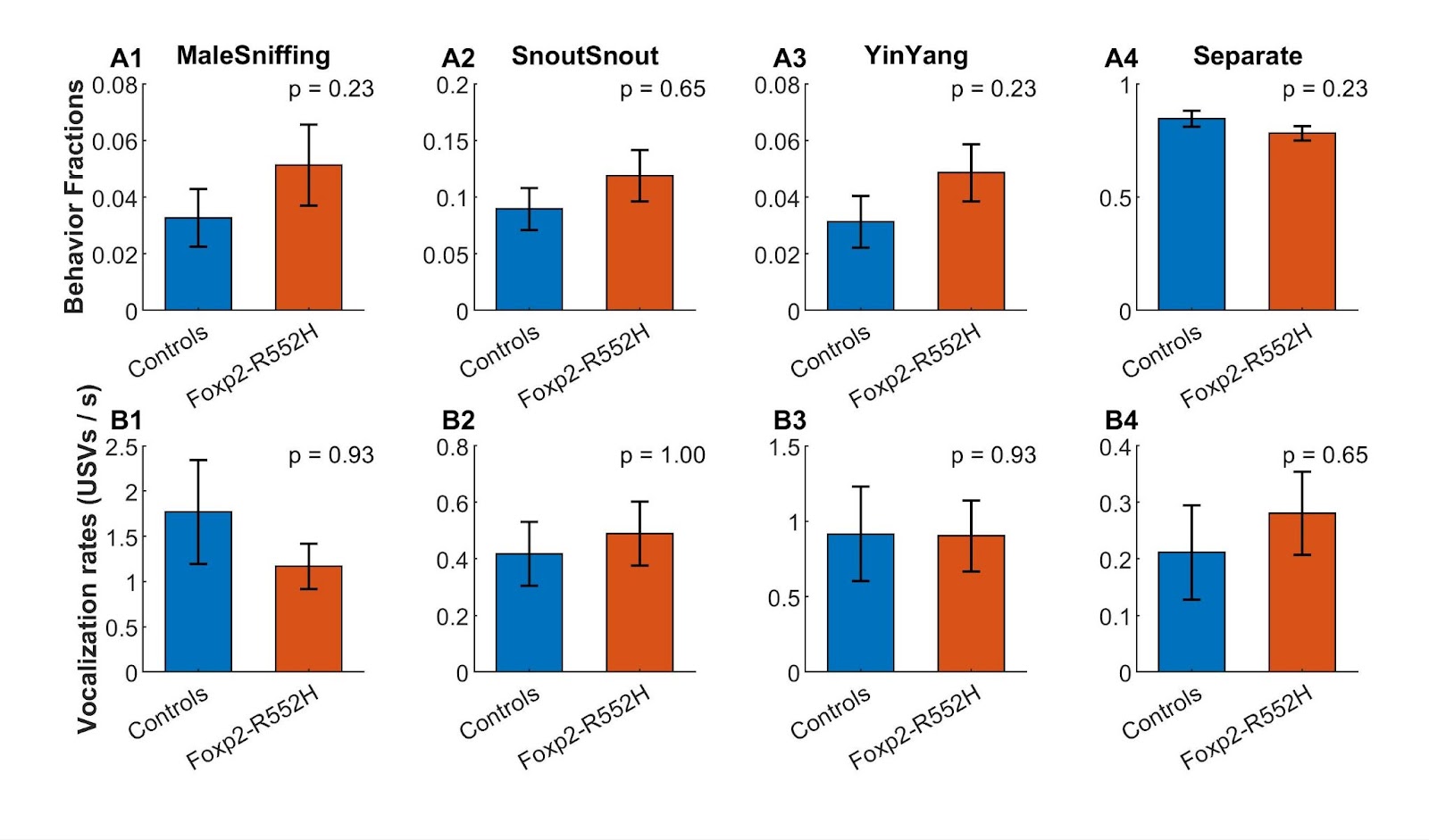
**

**Supplementary Figure 9:** Comparison of behavior fractions (A1-A4) and vocalization rates (B1-B4) within specific behaviors between male Foxp2-R552H and WT littermates. Behavior annotation performed by JAABA. Significance analysis conducted across mice (i.e. average taken per mouse, then comparison between individual mice). Wilcoxon rank sum test with Bonferroni correction accounting for multiple comparisons (𝛼 = 0.0125). Error bars depict standard errors. Note: the p-values in multiple cases are indeed identical, which can happen as the Wilcoxon rank sum test does not consider the actual values, but only their ranks.
