# Supplementary material for: High-precision spatial analysis of mouse courtship vocalization behavior reveals sex and strain differences
Source: Sci Rep. 2023 Mar 30;13:5219. doi: 10.1038/s41598-023-31554-3 (PMC10063627; doi:10.1038/s41598-023-31554-3)
Supplement: Supplementary file 15 — Supplementary Figure 10. [file 41598_2023_31554_MOESM15_ESM.docx]

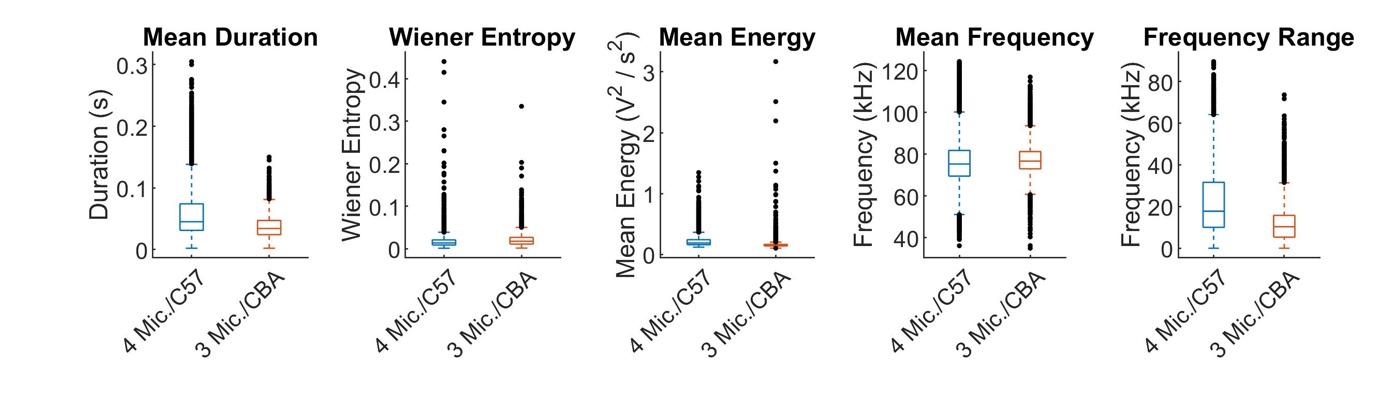


**Supplementary Figure 10:** Comparison of USV properties recorded with 4 microphones (C57BI/6J WT and Foxp2-R552H) and 3 microphones (CBA/CaJ).
